# A General 3D Model for Growth Dynamics of Sensory-Growth Systems: From Plants to Robotics

**DOI:** 10.3389/frobt.2020.00089

**Published:** 2020-08-05

**Authors:** Amir Porat, Fabio Tedone, Michele Palladino, Pierangelo Marcati, Yasmine Meroz

**Affiliations:** ^1^Faculty of Exact Sciences, School of Physics, Tel Aviv University, Tel Aviv, Israel; ^2^Gran Sasso Science Institute, L'Aquila, Italy; ^3^Faculty of Life Sciences, School of Plant Sciences and Food Security, Tel Aviv University, Tel Aviv, Israel

**Keywords:** plant tropism, circumnutation, self-growing robots, plant-inspired robotics, control system, optimal control, growth

## Abstract

In recent years, there has been a rise in interest in the development of self-growing robotics inspired by the moving-by-growing paradigm of plants. In particular, climbing plants capitalize on their slender structures to successfully negotiate unstructured environments while employing a combination of two classes of growth-driven movements: tropic responses, growing toward or away from an external stimulus, and inherent nastic movements, such as periodic circumnutations, which promote exploration. In order to emulate these complex growth dynamics in a 3D environment, a general and rigorous mathematical framework is required. Here, we develop a general 3D model for rod-like organs adopting the Frenet-Serret frame, providing a useful framework from the standpoint of robotics control. Differential growth drives the dynamics of the organ, governed by both internal and external cues while neglecting elastic responses. We describe the numerical method required to implement this model and perform numerical simulations of a number of key scenarios, showcasing the applicability of our model. In the case of responses to external stimuli, we consider a distant stimulus (such as sunlight and gravity), a point stimulus (a point light source), and a line stimulus that emulates twining of a climbing plant around a support. We also simulate circumnutations, the response to an internal oscillatory cue, associated with search processes. Lastly, we also demonstrate the superposition of the response to an external stimulus and circumnutations. In addition, we consider a simple example illustrating the possible use of an optimal control approach in order to recover tropic dynamics in a way that may be relevant for robotics use. In all, the model presented here is general and robust, paving the way for a deeper understanding of plant response dynamics and also for novel control systems for newly developed self-growing robots.

## 1. Introduction

Though the field of robotics has long been inspired from the capabilities of biological organisms, it is only recently that the plant world has become a source of inspiration, particularly due to the ability of plants to continuously change their morphology and functionality by growing, thus adapting to a changing environment (Del Dottore et al., 2016; Laschi and Mazzolai, 2016; Mazzolai et al., 2016). A new class of plant-inspired robots has emerged, based on the moving-by-growing capabilities of plants. Some recent examples include: (i) a tendril robot developed at NASA's Johnson Space Center, which is a slender manipulator composed of multiple bending segments (Mehling et al., 2006), (ii) a vine-bot that elongates its body at the tip by skin eversion, growing in a pre-determined form (Hawkes et al., 2017), (iii) plantoid robots inspired by plant roots, based on additive manufacturing technologies (Sadeghi et al., 2014, 2017; Del Dottore et al., 2016), and (iv) FIBERBOTS, based on the addition of fiber and resin (Kayser et al., 2019). Though these are impressive accomplishments, these robots are currently limited in their control systems and autonomy. The challenge lies in the fact that the morphology of such self-growing robots changes over time and is therefore not known in advance. Furthermore, in the future, such robots are expected to perform autonomously in unstructured scenarios, including achieving locomotion in uncertain terrains involving obstacles and voids, as well as the manipulation of unknown objects. Therefore, the development of a control system is not trivial and cannot be based on existing control systems of classic predefined robotic structures.

Plants, on the other hand, excel at these types of tasks. Though plants exhibit a variety of types of movements as part of their interaction with their environment (Darwin, 1880; Jost and Gibson, 1907; Ruhland, 1959; Hart, 1990; Forterre, 2013), here we focus on the relevant growth-driven movements of rod-like organs, such as shoots and roots. Such growth-driven movements are generally classified as either *nastic* or *tropic* (Rivière et al., 2017). Nastic movements are due to internal drivers, such as the inherent periodic movement of plants called circumnutations, sometimes associated with search processes. Tropisms are the growth-driven responses of a plant in the direction of a stimulus, such as a plant shoot growing toward a source of light or away from the direction of gravity (Darwin, 1880; Gilroy and Masson, 2007; Rivière et al., 2017). Tropic responses are based on three main processes: (i) sensing of a directional external stimulus by specialized biosensors, (ii) transduction of signals within the plant, leading to the redistribution of the growth hormone auxin, resulting in (iii) an anisotropic growth pattern that reorients the organ toward or away from a given stimulus.

In order to emulate these complex growth dynamics in a 3D environment in a way that is meaningful from the robotics standpoint, a general mathematical framework is required. Recently developed models of growth-driven plant dynamics are limited to specific aspects of tropisms or circumnutations. Bastien et al. have developed models for tropism in 2D, such as the AC (Bastien et al., 2013, 2015) and ACE (Bastien et al., 2014) models, addressing the influence of growth, and identifying the requirement of a *restoring force* called proprioception, whereby a plant can dampen the curving dynamics according to how curved it is (Bastien et al., 2013; Hamant and Moulia, 2016). Bressan et al. (2017) developed a model based on a similar formalism, but not accounting for growth explicitly as the driver of dynamics and achieving stable dynamics by controlling the growth-zone and sensitivity rather than proprioception. Another model focuses on circumnutations in 3D (Bastien and Meroz, 2016) but disregarding tropic responses. These models disregard elastic responses, implicitly assuming that no forces or torques act on the organs. Recently, efforts have been made to consider elastic responses in specific scenarios, namely incorporating gravitational forces in the case of gravitropism (Chelakkot and Mahadevan, 2017) and in the case of circumnutations (Agostinelli et al., 2020).

Here, we present a general and rigorous mathematical framework of a rod-like growing organ whose dynamics are driven by both internal and external cues. Though this model is inspired by plant responses, it is not based on biological details and is therefore relevant to any rod-like organisms that respond to signals via growth, such as neurons and fungi. The model does not include elastic responses, but the mathematical framework we adopt here allows a natural integration of elasticity, which we plan to do in future work. The paper is organized as follows: section 2 describes the dynamical equations of our model based on a 3D description of an organ in the Frenet-Serret formalism, implementing differential growth as the driver of movement, and relating external and internal signals. In section 3, we present the numerical method required to implement this model, and in section 4, we perform numerical simulations of a number of key case examples, including responses to external stimuli, such as a distant stimulus, a point stimulus, and a line stimulus, as well as circumnutations (the response to an internal oscillatory cue). We also present an example where we superimpose two different types of cues, namely the response to an external stimulus and circumnutations. Lastly, in section 5, we consider a simple example illustrating the possible use of an optimal control approach in order to recover tropic dynamics in a way which may be amenable to robotics use.

## 2. Governing Equations

In this section, we develop the dynamical equations that form the basis of our model. We first introduce a 3D description of an organ in the Frenet-Serret formalism and then detail the implementation of growth and differential growth as the driver of movement. Finally, we relate external and internal signals to differential growth, which drives the desired movement. We then show that our model is a generalization that consolidates different aspects of existing models, allowing the characteristic time and length scales of our model to be identified and discussed.

### 2.1. 3D Description of an Organ

**Figure 1:**
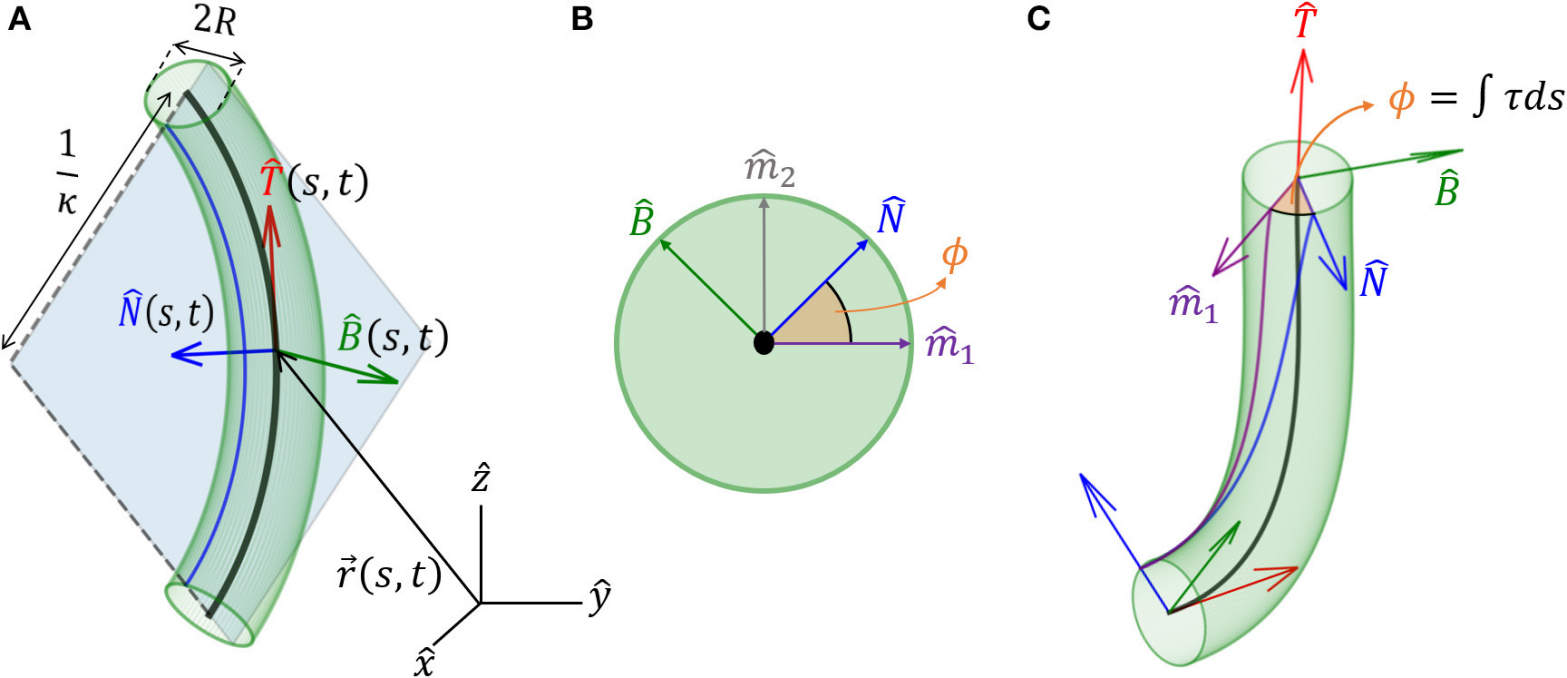
Geometrical definitions for a 3D cylindrical organ. **(A)** A cylindrical organ of constant radius *R* is described by its centerline, parameterized by the arc-length *s*. $\vec{r}\left(s,t\right)$ denotes the Cartesian position of a point along the centerline at point *s* and time *t*. The local Frenet-Serret frame at some point along the centerline is defined by the tangent vector $\hat{T}\left(s,t\right)=\partial \vec{r}\left(s,t\right)/\partial s$, its derivative the normal vector $\hat{N}\left(s,t\right)$ (Equation 2), and the bi-normal vector $\hat{B}\left(s,t\right)$ (Equation 3). Here, the organ has a constant curvature κ and is restricted to a plane, illustrating 1/κ(*s, t*) as the radius of curvature. **(B)** Cross-section of the organ and the *natural* frame: ($\hat{N}$, $\hat{B}$) span the cross-section, (${\hat{m}}_{1}$, ${\hat{m}}_{2}$) are constant vectors defining the natural frame, as described in section 3, and ϕ(*s, t*) defines the angle between $\hat{N}$ and the reference vector ${\hat{m}}_{1}$. **(C)** An organ not restricted to a plane. Here ϕ(*s, t*) changes along s, and torsion is defined as τ = ∂ϕ/∂*s*. Note that in **(A)**, τ = 0.

We model an elongated rod-like organ as a curved cylinder with radius *R*, described by its centerline that follows a curve in 3D. We denote the location of the centerline from the origin of a Cartesian frame of reference as $\vec{r}\left(s,t\right)$, where *t* is time, and *s* is its arc-length, which runs along the organ, taking the value *s* = 0 at the base and *s* = *L* at the apical tip, equal to the total length (see Figure 1A). In order to describe the dynamics of the centerline with respect to local stimuli, we begin by defining a local frame of reference using the Frenet-Serret framework (Goriely, 2017). Using the Frenet-Serret formulas for a 3D curve parameterization, as shown in Figure 1A, we can define the tangent vector at arc-length *s* as:

(1) $$\begin{array}{l} \hat{T}\left(s,t\right)=\frac{\partial }{\partial s}\vec{r}\left(s,t\right), \end{array}$$

where $\hat{T}$ is a unit vector, from the definition of the arc-length (Goriely, 2017). The second derivative of $\vec{r}$ can be written as:

(2) $$\begin{array}{l} \frac{\partial^{2}}{\partial s^{2}}\vec{r}\left(s,t\right)=\frac{\partial }{\partial s}\hat{T}\left(s,t\right)=\kappa \left(s,t\right)\hat{N}\left(s,t\right), \end{array}$$

where κ is the local curvature of the curve, and $\hat{N}$ is the respective normal vector. We note that when κ = 0, $\hat{N}$ is not defined, in which case we adopt a related local frame described in section 3. Since $|\frac{\partial }{\partial s}\vec{r}|=|\hat{T}|=1$, taking the derivative of $\hat{T}\cdot \hat{T}=1$ yields $2\hat{T}\cdot \frac{\partial \hat{T}}{\partial s}=0$, meaning that $\hat{T}⊥\frac{\partial \hat{T}}{\partial s}$, i.e., we have $\hat{T}⊥\hat{N}$. The curvature equals the inverse of the radius of curvature, and the normal vector $\hat{N}$ points to the center of the circle with that radius. The third unit vector in the Frenet-Serret framework is the bi-normal vector $\hat{B}\left(s,t\right)$, which creates an orthogonal basis in 3D, as illustrated in Figure 1A:

(3) $$\begin{array}{l} \hat{B}=\hat{T}\times \hat{N}. \end{array}$$

For the sake of legibility, we interchangeably omit writing the explicit dependence of variables on (*s, t*), i.e., when we write $\hat{T}$, we mean $\hat{T}\left(s,t\right)$.

The Frenet-Serret framework describes the change in this local frame of reference as a function of the arc-length *s* (Goriely, 2017):

(4) $$\begin{array}{l} \frac{\partial }{\partial s}\hat{T}=\kappa \hat{N}\\ \begin{array}{l} \frac{\partial }{\partial s}\hat{N}=-\kappa \hat{T}+\tau \hat{B} \end{array}\\ \begin{array}{l} \frac{\partial }{\partial s}\hat{B}=-\tau \hat{N}, \end{array} \end{array}$$

so that the local coordinate system changes accordingly along the curve. Here, κ(*s, t*) is the curvature and τ(*s, t*) is the torsion of the centerline, describing rotations in the ($\hat{N},\hat{B}$) plane leading to a non-planar centerline, as illustrated in Figures 1B,C. We now define ϕ, the angle between $\hat{N}$ and arbitrarily chosen fixed direction ${\hat{m}}_{1}$ (Bishop, 1975; Langer and Singer, 1996; Bastien and Meroz, 2016). The change in the direction of $\hat{N}$ along the curve yields the torsion τ(*s, t*) (see Figure 1C):

(5) $$\begin{array}{l} \tau \left(s,t\right)=\frac{\partial }{\partial s}\phi \left(s,t\right). \end{array}$$

### 2.2. Modeling Growth and Differential Growth

**Figure 2:**
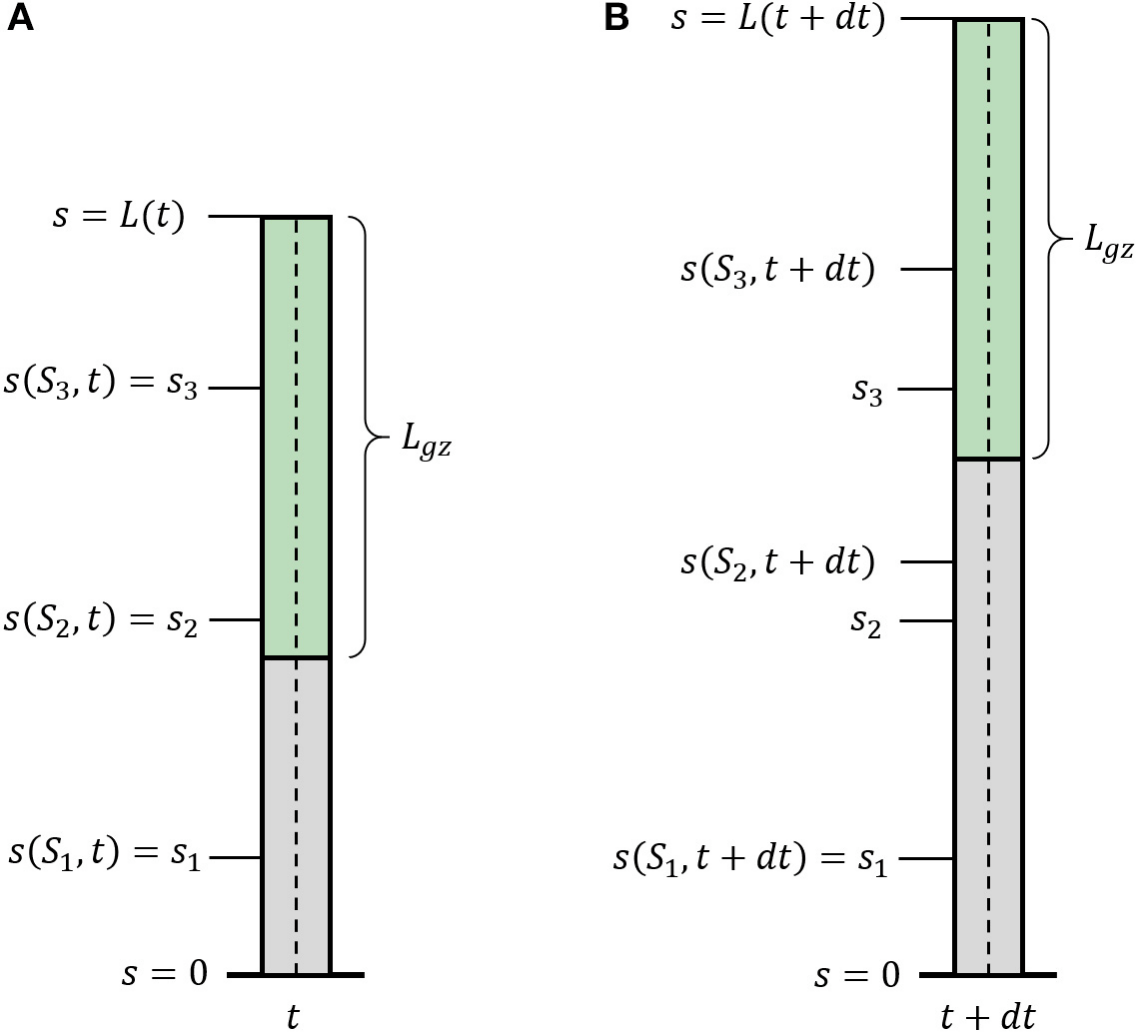
Growth description. Illustration of a growing organ with a sub-apical growth zone, marked in green. The centerline (dashed line) can be parameterized by a material coordinate, *S*_0_, or by the arc-length, *s*(*S*_0_, *t*). **(A)** The organ at time *t*; **(B)** the organ at time *t* + *dt*. Outside of the growth zone, the position of the material coordinate does not change in time *s*(*S*_1_, *t* + *dt*) = *s*(*S*_1_, *t*). Within the growth zone *s*(*S*_3_, *t* + *dt*) > *s*(*S*_3_, *t*), i.e., the location of the material coordinated flows due to growth, and *S*_2_ and *S*_3_ flow within the organ. *S*_2_ flows out of the growth zone and will stay fixed.

We now introduce growth, using similar definitions to those introduced in Silk (1989), Bastien et al. (2014), and Goriely (2017). We define *S*_0_ as the arc-length of the initial centerline of the organ, and the current arc length *s*(*S*_0_, *t*) as the evolution of the point *S*_0_ in time, with initial conditions *s*(*S*_0_, *t* = 0) = *S*_0_. One can think of the arc-length *s*(*S*_0_, *t*) as describing the flow of the initial point *S*_0_ due to the growth of all previous parts of the organ (see Figure 2). Therefore, assuming that the organ does not shrink, *s*(*S*_0_, *t*) monotonically increases over time. This growth-induced flow within the organ motivates us to use definitions from fluid dynamics, in which the parameter *S*_0_ can be thought of as the Lagrangian, referential, or material coordinate and *s* as the Eulerian or spatial coordinate (Goriely, 2017). Using regular conventions of continuum mechanics, we define the local velocity of point *s* as the accumulation of the growth that occurs in previous parts of the organ:

(6) $$\begin{array}{l} \frac{ds}{dt}=v\left(s,t\right)=\int_{0}^{s}\dot{E}\left(u,t\right)du, \end{array}$$

where $\dot{E}$(*s, t*) is the local growth rate, representing a combination of the effect of the addition of new cells and their elongation. We define the length of the active growth-zone of a growing organ *L*_*gz*_ as the length over which the growth rate $\dot{E}$(*s, t*) is non-zero. Without loss of generality, here we will consider the common case where growth is confined to a finite sub-apical growth-zone: *L* − *L*_gz_ ≤ *s* ≤ *L*, as shown in Figure 2. However, the growth zone may be defined along any other relevant section of the organ, for example when considering internodal growth. We note that as opposed to Lagrangian quantities (functions of *S*_0_), the time derivative of Eulerian fields [functions of *s*(*S*_0_, *t*)] incurs an additional convection term. We use the convention of material derivatives for the total time derivative, namely: *D*/*Dt* ≡ ∂/∂*t* + *v∂*/∂*s*.

As mentioned in the Introduction, plant tropisms are the growth-driven reorientation of plant organs due to a directional stimulus, such as light, gravity, or water gradient. In particular, the reorientation of the plant organ is due to differential growth, i.e., one side of the cylindrical organ grows at a higher rate than the other side, resulting in a curved organ. Following Bastien and Meroz (2016), we consider an infinitesimal cross-section of a cylindrical organ and define the differential growth rate in a direction $\hat{e}$ as the difference in growth rate $\dot{\epsilon }$ on either side, normalized by their sum:

(7) $$\begin{array}{l} \Delta \left(\hat{e}\right)=\frac{\dot{\epsilon }\left(-\hat{e}\right)-\dot{\epsilon }\left(\hat{e}\right)}{\dot{\epsilon }\left(-\hat{e}\right)+\dot{\epsilon }\left(\hat{e}\right)}. \end{array}$$

**Figure 3:**
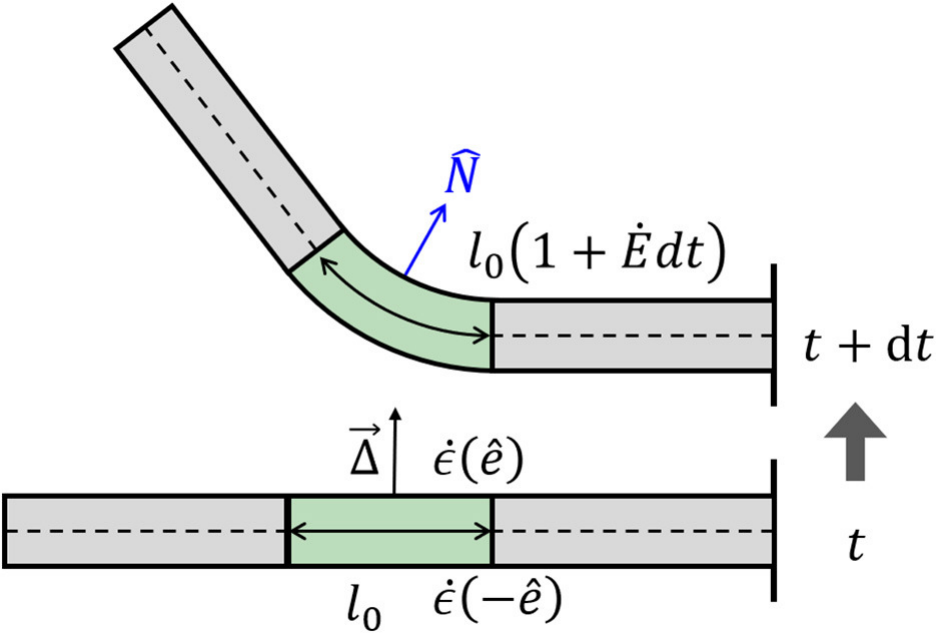
Differential growth. Differences in growth rates across a cylinder lead to a change in curvature. At time *t*, we have a straight organ with κ(*t*) = 0 and with a growth zone in the center of length *l*(*t*) = *l*_0_, marked in green. The differential growth vector $\vec{\Delta }$ in the growth zone is constant and points upwards in the $\hat{e}$ direction. Following Equation (7), the growth rate on the lower side is higher than that in the upper side $\dot{\epsilon }\left(-\hat{e}\right)<\dot{\epsilon }\left(\hat{e}\right)$, and after a time interval *dt*, the two sides grow different amounts, leading to bending of the growth zone with a new curvature κ(*t* + *dt*) > 0. The new length of the growth zone along the centerline is now *l*(*t* + *dt*) = *l*_0_(1 + $\dot{E}$*dt*). Note that changes in curvature in the middle of the organ lead to changes in orientation of the rest of the organ.

Following this definition, for Δ($\hat{e}$) > 0, the organ grows faster in direction −$\hat{e}$ and the organ bends in direction $\hat{e}$ (see Figure 3). We now define the differential growth vector, which is in the direction of the active reorientation:

(8) $$\begin{array}{l} \vec{\Delta }=\Delta \left(\hat{N}\right)\hat{N}+\Delta \left(\hat{B}\right)\hat{B}. \end{array}$$

In order to describe the active reorientation of an entire organ, we relate the shape of the organ and its growth dynamics, expressed by the dynamics of its local curvature, $D\left(\kappa \hat{N}\right)/Dt$, to the differential growth term $\vec{\Delta }$ (Bastien and Meroz, 2016), resulting in:

(9) $$\begin{array}{l} \begin{array}{c} \frac{D}{Dt}\kappa =\frac{\dot{E}}{R}\vec{\Delta }\cdot \hat{N}\\ \kappa \frac{D}{Dt}\phi =\frac{\dot{E}}{R}\vec{\Delta }\cdot \hat{B}, \end{array} \end{array}$$

where the equations have been linearized by assuming that the radius of curvature 1/κ is always larger than the radius of the organ (κ*R* ≪ 1). For a detailed calculation, see Appendix A. These equations are similar to those developed in (Bastien and Meroz, 2016), where the differential growth vector represented the internal cues related to circumnutations. Given an expression for the differential growth vector $\vec{\Delta }\left(s,t\right)$ and an initial configuration, the dynamics can be integrated completely. The form of $\vec{\Delta }\left(s,t\right)$ is dictated by either internal cues (circumnutations) or external stimuli, as discussed in the following section.

### 2.3. Relating External and Internal Signals to Differential Growth

In the last section, we represented the anisotropic growth pattern by the local differential growth vector $\vec{\Delta }\left(s,t\right)$. An external signal is translated to a specific growth pattern thanks to signal-specific biosensors and biochemical signal transduction mechanisms. Here, we reduce these complex processes to a sensitivity or gain function that maps the external signal to a growth response and directly governs the differential growth vector.

**Figure 4:**
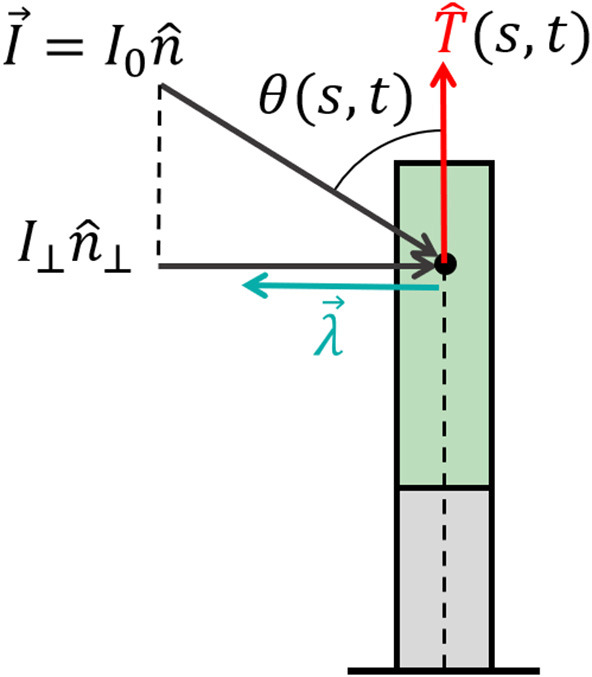
Effective signal and response vector. An example of a signal that can be described by a constant vector field (such as sunlight and gravity) of the form $\vec{I}=I_{0}\hat{n}$, where *I*_0_ is the magnitude of the stimulus and $\hat{n}$ is its direction. For an element of an organ, the relevant directional information of the stimulus lies within its projection perpendicular to the organ surface, which we define as ${\vec{I}}_{⊥}=I_{⊥}{\hat{n}}_{⊥}$ (see Equation 10), where the magnitude is given by *I*_⊥_ = *I*_0_ sin (θ(*s, t*)) and θ(*s, t*) is the angle between the surface $\hat{T}$ and $\hat{n}$. Biophysical laws generally describe sensory responses to input signals as functions of the signal intensity λ(*I*) (see main text). We define the response vector $\vec{\lambda }\left(s,t\right)=-\lambda \left(I_{⊥}\left(s,t\right)\right){\hat{n}}_{⊥}\left(s,t\right)$ where the magnitude of the perceived response is given by the sensitivity function λ(*I*_⊥_(*s, t*)), and ${\hat{n}}_{⊥}\left(s,t\right)$ is the direction of the effective stimulus.

Environmental signals can be mathematically described as fields. For example, vector fields describe light and gravity, while a scalar field describes the concentration of water or nutrients, and the direction of increasing concentrations is again described by a vector field of the gradients. Lastly, tensor fields may describe stress and strain; however, we will not discuss these here since our model does not include elasticity. Here we focus on vector fields, where we can write the directional stimulus in the form $\vec{I}\left(\vec{r}\right)\equiv I\left(\vec{r}\right)\hat{n}\left(\vec{r}\right)$, where $I\left(\vec{r}\right)$ is the magnitude of the stimulus at a point $\vec{r}$ in space, and $\hat{n}\left(\vec{r}\right)$ is its direction. For example, in the case of an infinitely distant stimulus, such as light or gravity, the stimulus magnitude and direction are constant in space, i.e., $\vec{I}\left(\vec{r}\right)=I_{0}\hat{n}$. In the case of a chemical concentration gradient, a possible form would be $\vec{I}\left(\vec{r}\right)=\vec{\nabla }c\left(\vec{r}\right)$, though the sensed magnitude may depend on other factors, such as the concentration itself and remains to be verified. The physics of the signal and the geometry of the emitting source dictate the direction of the stimulus $\hat{n}\left(\vec{r}\right)$. Within a specific infinitesimal element of an organ, the differential growth vector is restricted to the cross-section, i.e., the local $\left(\hat{N},\hat{B}\right)$ plane. Therefore, the relevant directional information of the stimulus lies within its projection perpendicular to the organ surface, as illustrated in Figure 4. We define the component of the stimulus perpendicular to this surface, ${\vec{I}}_{⊥}\left(s,t\right)$, as the effective stimulus sensed by the organ. From geometrical arguments, assuming that the stimulus field changes slowly around the cross-section of the organ, the effective stimulus sensed by a cylindrical surface is given by:

(10) $$\begin{array}{l} {\vec{I}}_{⊥}=\hat{T}\times \left(\vec{I}\times \hat{T}\right)\equiv I_{⊥}{\hat{n}}_{⊥}, \end{array}$$

where we have defined ${\hat{n}}_{⊥}\left(s,t\right)$ as the direction of the perpendicular component of the signal and *I*_⊥_(*s, t*) as its magnitude given by $I_{⊥}=I\left(\vec{r}\right)\text{sin }\left(\theta \left(s,t\right)\right)$, where θ(*s, t*) is the angle between the surface $\hat{T}\left(s,t\right)$ and the direction of the stimulus $\hat{n}\left(s,t\right)$, as shown in Figure 4.

Two central biophysical laws describe sensory responses to input signals, which we term here the sensitivity function λ(*I*). One is a logarithmic relationship λ(*I*) = *a* + *b*log(*I*/*I*_0_), referred to as the Weber-Fechner law (Norwich and Wong, 1997), and the other is a power law relationship λ(*I*) = *aI*^*b*^, known as Stevens' law (Stevens, 1957). As an example it has been found that phototropism follows Stevens' Law (Bastien et al., 2015), while in the case of gravitropism, only inclination is sensed, and sensitivity is constant, λ(*g*) = const (Chauvet et al., 2016). However, very little is known for other plant tropisms. We now define the local response vector:

(11) $$\begin{array}{l} \vec{\lambda }\left(s,t\right)=-\lambda \left(I_{⊥}\left(s,t\right)\right){\hat{n}}_{⊥}\left(s,t\right), \end{array}$$

where the sensitivity function takes the effective stimulus sensed by the organ λ(*I*_⊥_(*s, t*)), and ${\hat{n}}_{⊥}\left(s,t\right)$ is the direction of the effective stimulus. As stated before, the differential growth vector is restricted to the cross-section plane of the organ element, and it is therefore directly related to the perpendicular component of the stimulus field and its response vector, i.e., $\vec{\Delta }\left(s,t\right)=\vec{\lambda }\left(s,t\right)$.

However, it has been found that a so-called *restoring force* is required for stable posture control, termed *proprioception* (Bastien et al., 2013; Hamant and Moulia, 2016). This is related to an internal process associated with the active tendency of a growing organ to resist being bent (not a mechanical response), and is represented by $-\gamma \kappa \left(s,t\right)\hat{N}\left(s,t\right)$, where γ is the proprioceptive sensitivity (Bastien et al., 2013). We also note that differential growth may be due to internal processes, such as in the case of circumnutations. Here, an internal oscillator turns the differential growth vector in the ($\hat{N},\hat{B}$) plane (Bastien and Meroz, 2016) and can be described as $\vec{\chi }\left(s,t\right)=\lambda_{0}\left(\text{cos }\left(\psi \left(t\right)\right){\hat{m}}_{1}\left(s,t\right)+\text{sin }\left(\psi \left(t\right)\right){\hat{m}}_{2}\left(s,t\right)\right)$, where λ_0_ is the intensity of the bending, and ψ(*t*) is a general function describing the direction of growth at time *t* relative to fixed vectors $\left({\hat{m}}_{1},{\hat{m}}_{2}\right)$ (Figure 1B). Here, we chose a circular growth pattern; however, more elaborate forms can be implemented (Bastien and Meroz, 2016). Assuming that the different mechanisms are additive, and adding the propriocetion term and circumnutations, the differential growth vector therefore follows:

(12) $$\begin{array}{l} \vec{\Delta }\left(s,t\right)=\vec{\lambda }\left(s,t\right)+\vec{\chi }\left(s,t\right)-\gamma \kappa \hat{N}\left(s,t\right). \end{array}$$

**Figure 5:**
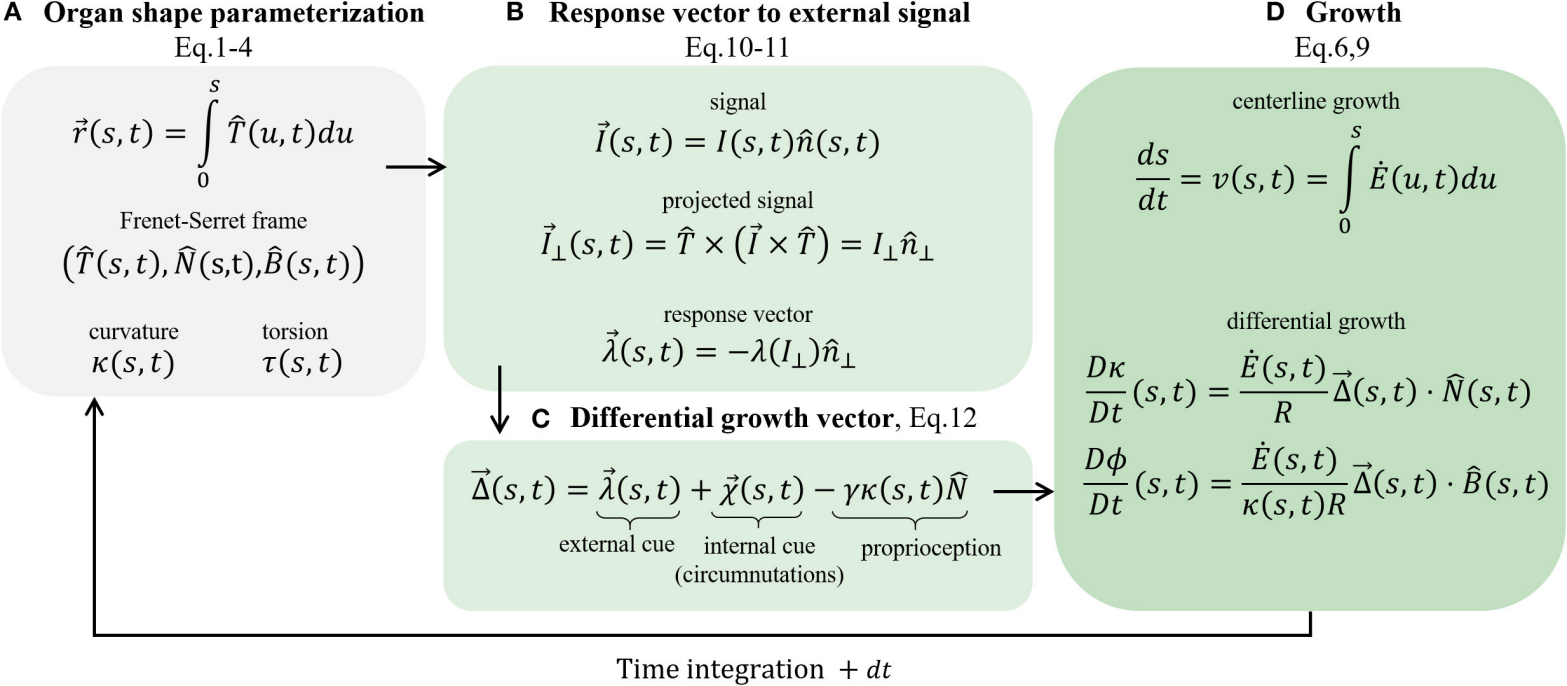
Schematic of the governing equations. We present the main stages involved in the model. **(A)** Organ shape parametrization, section 2.1: the Frenet Serret local frame in Equations (1)–(3), and Frenet-Serret equations that also define κ and τ, introduced in Equation (4). **(B)** Response vector to external signal, section 2.3: assuming a vector field signal, we find the projected signal (Equation 10) and calculate the response vector (Equation 11), which affects the growth response. **(C)** Differential growth vector (Equation 12); includes terms representing external cues (the response vector), internal cues (circumnutations), and proprioception for posture control. **(D)** Implementing growth dynamics, section 2.2. The centerline is updated using Equations (6) and (9), using the constructed differential growth vector.

Together with Equation (9), Equation (12) completes our model for active growth-driven movements of rod-like organs in 3D taking into account external signals, internal cues (circumnutations), and posture control. For multiple stimuli, again assuming additivity, one can replace $\vec{\lambda }$ with the sum of specific response vectors $\underset{m}{\sum }{\vec{\lambda }}_{m}$ (Bastien et al., 2015). A number of specific cases, including various types of external and internal cues, are explained in further detail in section 4. A schematic summarizing the governing equations is presented in Figure 5.

Lastly, the distribution of sensory systems along the organ also requires attention. Sensory systems in plant organs are generally either distributed along the organ, providing local sensing (Sakamoto and Briggs, 2002; Wan et al., 2008; Hohm et al., 2013), or restricted to the tip, termed apical sensing (Darwin, 1880; Knieb et al., 2004; Holland et al., 2009; Hohm et al., 2013). For example, in the case of shoot phototropism, photoreceptors are localized at the tip alone, such as in wheat, or distributed along the whole growth zone, as in the case of Arabidopsis. In the case of gravitropism, specialized cells called statocytes sense the direction of gravity, and these are generally found throughout the growth zone for aerial organs and restricted to the tip for roots (Morita and Tasaka, 2004; Su et al., 2017). In the case of apical sensing, the local response vector $\vec{\lambda }\left(s,t\right)$ will be replaced with that of the apex $\vec{\lambda }\left(L,t\right)$ rotated to the local frame, meaning that the whole organ responds to what is sensed at the tip alone.

### 2.4. Comparison to Previous Models

Different models of growth-driven plant dynamics have been recently developed, encompassing different aspects of tropisms and circumnutations. Bastien et al. (2013, 2014, 2015) have developed a model for tropism in 2D, addressing the influence of growth and identifying the requirement of proprioception. A third model (Bressan et al., 2017) is based on a similar formalism, achieving stable dynamics by controlling the growth-zone and sensitivity rather than proprioception. Another model focuses on circumnutations in 3D (Bastien and Meroz, 2016), disregarding tropic responses. In what follows, we show how our model relates to these previous models while also generalizing them and unifying them.

In order to compare with 2D models, we focus on the case where the dynamics of our model are restricted to a 2D plane, which occurs when the direction of the stimulus $\hat{n}$ is in the plane defined by $\hat{T}$ and $\hat{N}$. In this case, following Equation (12), only the component in the $\hat{N}$ direction of $\vec{\Delta }$ is not zero. Substituting this into the dynamical equations in Equation (9), since $\vec{\Delta }$$\cdot \hat{B}=0$, we get that *Dϕ*/*Dt* = 0, i.e., ϕ is constant. Assuming an initially straight organ, ϕ = 0 throughout, yielding τ = ∂ϕ/∂*s* = 0. The geometrical meaning is that when the stimulus and the initial state of the organ are in the same 2D plane, the dynamics of the organ will remain within that plane and therefore restricted to 2D. In this case, we can compare the dynamics directly to Bastien et al. (2015) by projecting the model to 2D, and assuming a constant signal. We define θ the local angle of $\hat{T}$ along the organ with respect to the direction of the constant signal $\hat{n}$, as illustrated in Figure 4, i.e., $\theta \left(s,t\right)=\text{arccos}\left(\hat{T}\left(s,t\right)\cdot \hat{n}\right)$. Taking the derivative over the arc-length *s* and recalling that $\partial \hat{T}/\partial s=\kappa \hat{N}$ (Equation 4) yields: $\frac{\partial }{\partial s}\theta \left(s,t\right)=\pm \kappa \left(s,t\right)$, where the sign depends on the direction of $\hat{N}$. Substituting these expressions into Equations (9) and (12), together with $\hat{n}\cdot \hat{N}=-\text{sin }\left(\theta \right)$ and a constant sensitivity function λ(*I*) = λ, we get:

(13) $$\begin{array}{l} \frac{D}{Dt}\kappa \left(s,t\right)=\frac{\dot{E}}{R}\left(-\lambda \text{sin }\theta \left(s,t\right)-\gamma \kappa \left(s,t\right)\right), \end{array}$$

identical to the ACE model developed in Bastien et al. (2014).

We now consider Bressan et al. (2017). Their main equation of motion appears in Equation (2.8), and translating this into our terminology takes the form:

(14) $$\begin{array}{l} \frac{\partial }{\partial t}\hat{T}=\left(\int_{0}^{s}\lambda e^{-\eta \left(t-\sigma \right)}\left(\hat{T}\times \hat{n}\right)d\sigma \right)\times \hat{T}, \end{array}$$

where λ > 0 is a constant measuring the strength of the response, similar to our tropic sensitivity, while *e*^−η(*t*−σ)^ is what they call a stiffness factor. The simplest way to compare with this model is by looking at its 2D projection. Taking $\hat{T}=\left(\text{sin }\theta \left(s,t\right),\text{cos }\theta \left(s,t\right)\right)$, where θ(*s, t*) is the angle between $\hat{T}$ and $\hat{n}$, and substituting this in Equation (14) leads to $\frac{\partial }{\partial t}\theta \left(s,t\right)=-\int_{0}^{s}\lambda e^{-\eta \left(t-\sigma \right)}\text{sin }\theta \left(\sigma ,t\right)d\sigma$. Taking a derivative in s finally yields:

(15) $$\begin{array}{l} \frac{\partial }{\partial t}\kappa \left(s,t\right)=-\lambda e^{-\eta \left(t-s\right)}\text{sin }\theta \left(s,t\right). \end{array}$$

We note that this model considers accretive growth, where material is added at the tip, and elongation is disregarded. This means that growth is only taken into account implicitly as the driver of the tropic movement, and a material derivative is not required, which is a good approximation of the dynamics in certain cases (Bastien et al., 2013, 2014). In this case, the ACE model in Equation (13) converts to the AC model:

(16) $$\begin{array}{l} \frac{\partial }{\partial t}\kappa \left(s,t\right)=-\lambda \text{ sin }\theta \left(s,t\right)-\gamma \kappa \left(s,t\right). \end{array}$$

Comparing Equations (15) and (16), we see that the equations are similar: the response, appearing on the l.h.s., is identical, and on the r.h.s., the tropic stimulus is represented by sinθ(*s, t*) in both, as well as a sensitivity factor. In Bressan's model the stiffness prefactor *e*^−η(*t*−*s*)^ represents a smooth growth zone with a characteristic size of 1/η: in the youngest parts (s=t at the tip) the stiffness factor is 1, while in older parts of the organ (as s goes to zero), the stiffness factor goes to 0. We also notice that Bressan et al. do not use a proprioceptive term, generally required for stable dynamics; however, they were able to circumvent this problem by using small growth zones.

### 2.5. Characteristic Length and Time Scales

In section 2.4, we show that in the case where the dynamics of our model are restricted to a 2D plane, our model recovers the ACE model developed by Bastien et al. (2014). Thanks to this relation, we can adopt their dimensional analysis (Bastien et al., 2013), which identifies characteristic length and time scales. Consider the case of a constant stimulus placed perpendicular to a shoot. The length scale is identified by considering the steady state, where the shoot has grown in the direction of the stimulus, achieving a steady-state form, with $\frac{D\kappa \left(s,t\right)}{Dt}=0$ everywhere, including the growth zone. Substituting this into Equation (13) yields the maximal curvature value κ_max_, and its inverse, the radius of curvature, corresponds to a characteristic length scale termed the *convergence length L*_*c*_ = 1/κ_max_ = γ/λ, where γ and λ are the proprioceptive and tropic sensitivities, respectively. There are two time scales. One is associated with the time it takes for the organ to reach its steady state, termed the *convergence time* and defined as *T*_*c*_ = *R*/$\dot{E}$γ. The other is associated with the time it takes the organ to align in the direction of the stimulus for the first time, termed the *arrival time*, defined as *T*_*v*_ = *R*/$\dot{E}$*L*_gz_λ. The ratio between the convergence length *L*_*c*_ and the length of the growth zone *L*_gz_, as well as the ratio between the convergence time *T*_*c*_ and arrival time *T*_*v*_, introduces a dimensionless number *B*, termed the *balance number* (Bastien et al., 2013; Hamant and Moulia, 2016), which describes the balance between the sensitivity to external stimuli and proprioception and is linearly related to the maximal curvature:

(17) $$\begin{array}{l} B\equiv \frac{L_{\text{gz}}}{L_{c}}=\frac{T_{c}}{T_{v}}=\frac{\lambda L_{\text{gz}}}{\gamma }=\kappa_{\text{max}}L_{\text{gz}}. \end{array}$$

Low values of *B* mean that *L*_*c*_ > *L*_gz_, i.e., the growth zone is not big enough to contain the full arc-length associated with bending toward the stimulus with a given curvature, or alternatively that *T*_*v*_ > *T*_*c*_, i.e., the organ dynamics converge before it is able to arrive to the desired orientation in the direction of the stimulus. High values of *B* mean that *L*_*c*_ < *L*_gz_, i.e., the growth zone can contain the full bending, or alternatively that *T*_*v*_ < *T*_*c*_, i.e., the organ arrives at the desired orientation before the dynamics converge, therefore also exhibiting damped oscillations. In other words, we see that the balance number *B* represents a relation between the final shape of the organ in steady state and the dynamics.

## 3. Numerical Method

### 3.1. Natural Frame for the Numerical Scheme

As stated in section 2, our model for active growth-driven dynamics, described by Equations (9) and (12) and schematically illustrated in Figure 5, is formulated in the Frenet-Serret frame. The Frenet-Serret frame is a natural choice to describe curves since the second derivative gives the local curvature, $\frac{\partial^{2}}{\partial s^{2}}\vec{r}=\kappa \hat{N}$, a natural geometrical quantity. However, within this framework, $\hat{N}$ is not defined when κ = 0. In order to avoid related numerical issues, in the numerical scheme, we adopt a related local frame termed the “natural frame” or the “normal development” (Bishop, 1975; Langer and Singer, 1996): assuming $\vec{r}\left(s,t\right)$ is a point along the centerline of the organ, the natural frame is described by the orthonormal vectors $\left(\hat{T}\left(s,t\right),{\hat{m}}_{1}\left(s,t\right),{\hat{m}}_{2}\left(s,t\right)\right)$, where $\hat{T}\left(s,t\right)=\frac{\partial }{\partial s}\vec{r}\left(s,t\right)$ is the tangent vector in Equation (1). The other two orthogonal vectors $\left({\hat{m}}_{1},{\hat{m}}_{2}\right)$ span the cross-section plane spanned by $\left(\hat{N},\hat{B}\right)$ in the Frenet Serret frame. The rotations of this local frame with respect to the arc length of the curve are described using the following equations, similar to the Frenet-Serret equations in (Equation 4):

(18) $$\begin{array}{l} \frac{\partial }{\partial s}\hat{T}=\kappa_{1}{\hat{m}}_{1}+\kappa_{2}{\hat{m}}_{2} \end{array}$$

(19) $$\begin{array}{l} \frac{\partial }{\partial s}{\hat{m}}_{1}=-\kappa_{1}\hat{T} \end{array}$$

(20) $$\begin{array}{l} \frac{\partial }{\partial s}{\hat{m}}_{2}=-\kappa_{2}\hat{T} \end{array}$$

Here, κ_1_(*s, t*) and κ_2_(*s, t*) are the curvature components of the local cross-section plane, and the total curvature κ(*s, t*) and torsion τ(*s, t*) are given by the relations:

(21) $$\begin{array}{l} \kappa =\sqrt{\kappa_{1}^{2}+\kappa_{2}^{2}}, \end{array}$$

(22) $$\begin{array}{l} \phi =\text{arctan}\left(\frac{\kappa_{2}}{\kappa_{1}}\right), \end{array}$$

(23) $$\begin{array}{l} \tau =\frac{\partial }{\partial s}\phi , \end{array}$$

where ϕ is the angle between $\hat{N}$ (in the Frenet-Serret frame) and ${\hat{m}}_{1}$, illustrated in Figure 1B, and is used to define τ in Equation (5). This frame is closely related to the Frenet-Serret frame; however, the cross-section directions $\left({\hat{m}}_{1},{\hat{m}}_{2}\right)$ are always well-defined, even when κ = 0. Within this frame, Equation (9) can be rewritten as (see Appendix A for a detailed calculation):

(24) $$\begin{array}{l} \frac{D}{Dt}\kappa_{1}=\frac{\dot{E}}{R}\vec{\Delta }\cdot {\hat{m}}_{1} \end{array}$$

(25) $$\begin{array}{l} \frac{D}{Dt}\kappa_{2}=\frac{\dot{E}}{R}\vec{\Delta }\cdot {\hat{m}}_{2} \end{array}$$

In order to solve the dynamics, we integrate Equations (24) and (25).

### 3.2. Discretization and Integration

The organ is divided into segments of length *ds*, and we rewrite functions of the centerline in a discrete form, following the general form:

(26) $$\begin{array}{l} X\left(s,t\right)\to X\left(n\cdot ds,m\cdot dt\right)\equiv X\left(n,m\right). \end{array}$$

We describe the location of the organ using the local coordinate system:

(27) $$\begin{array}{l} \vec{r}\left(N,m\right)=\overset{N}{\underset{n=0}{\sum }}\hat{T}\left(n,m\right)ds. \end{array}$$

The dynamics of the organ is described through the evolution of the local coordinate system. We rewrite Equations (18)–(20) in matrix form, which describe the change in the local frame of reference as a function of *s*:

(28) $$\begin{array}{l} \frac{\partial }{\partial s}\text{D}\left(n,m\right)=\text{U}\left(n,m\right)\text{D}\left(n,m\right), \end{array}$$

where D(*n, m*) is the rotation matrix:

(29) $$\begin{array}{l} \text{D}\left(n,m\right)=\left({\hat{m}}_{1}\left(n,m\right),{\hat{m}}_{2}\left(n,m\right),\hat{T}\left(n,m\right)\right), \end{array}$$

and U(*n, m*) is the skew symmetric Darboux matrix:

(30) $$\begin{array}{l} \text{U}\left(n,m\right)=\left(\begin{array}{ccc} 0 & 0 & -\kappa_{1}\left(n,m\right)\\ 0 & 0 & -\kappa_{2}\left(n,m\right)\\ \kappa_{1}\left(n,m\right) & \kappa_{2}\left(n,m\right) & 0 \end{array}\right). \end{array}$$

In order to integrate Equation (28) while keeping the orthonormality of the local frame, we take inspiration from Gazzola et al. (2018), relating the consecutive discrete matrices D(*n* + 1, *m*) and D(*n, m*) via a rotation matrix R(*n, m*):

(31) $$\begin{array}{l} \text{D}\left(n+1,m\right)=\text{R}\left(n,m\right)\text{D}\left(n,m\right). \end{array}$$

Since U(*n, m*) in Equation (28) is skew-symmetric, we use Rodrigues' rotation formula and the exponential map to express matrix R(*n, m*):

(32) $$\begin{array}{l} \text{R}\left(n,m\right)=\text{exp }\left(\text{U}\left(n,m\right)ds\right) \end{array}$$

This can be interpreted as a rotation around the axis $\vec{u}=\frac{\kappa_{2}}{\kappa }{\hat{m}}_{1}-\frac{\kappa_{1}}{\kappa }{\hat{m}}_{2}$ by an angle κ(*n, m*)*ds* (or as the identity matrix for κ(*n, m*) = 0). It is therefore enough to find the evolution of U or the evolution of κ_1_ and κ_2_ to describe the organ in time. To integrate κ_1_ and κ_2_, we discretize Equations (24) and (25), adopting the following numerical time and arc-length derivatives (where *dt* is the discretized time step):

(33) $$\begin{array}{l} \dot{X}\left(n,m\right)=\frac{X\left(n,m+1\right)-X\left(n,m\right)}{dt} \end{array}$$

(34) $$\begin{array}{l} X^{\prime }\left(n,m\right)=\frac{X\left(n,m\right)-X\left(n-1,m\right)}{ds}, \end{array}$$

leading to:

(35) $$\begin{array}{l} {\dot{\kappa }}_{1}\left(n,m\right)+v\left(n,m\right)\frac{\kappa_{1}\left(n,m\right)-\kappa_{1}\left(n-1,m\right)}{ds}=\frac{\dot{E}}{R}\vec{\Delta }\left(n,m\right)\cdot \\ \begin{array}{l} {\;\hat{m}}_{1}\left(n,m\right) \end{array} \end{array}$$

(36) $$\begin{array}{l} {\dot{\kappa }}_{2}\left(n,m\right)+v\left(n,m\right)\frac{\kappa_{2}\left(n,m\right)-\kappa_{2}\left(n-1,m\right)}{ds}=\frac{\dot{E}}{R}\vec{\Delta }\left(n,m\right)\cdot \\ \begin{array}{l} {\;\hat{m}}_{2}\left(n,m\right). \end{array} \end{array}$$

The growth speed appearing in the material derivative, *v*(*n, m*), is calculated following Equation (6). Assuming a growth-zone of length *L*_gz_ and uniform growth rate $\dot{E}$ leads to:

(37) $$\begin{array}{l} v\left(n,m\right)=\left(L_{\text{gz}}-\left(N\left(m\right)-n\right)ds\right)\dot{E} \end{array}$$

in the case *L*_gz_ ≥ (*N*(m) − *n*)*ds*, and *v*(*n, m*) = 0 otherwise. Extracting ${\dot{\kappa }}_{1}$ and ${\dot{\kappa }}_{2}$ from Equations (35) and (36), we substitute these into Equation (33). Together with the following straight initial conditions and clamped boundary conditions of the organ, we integrate over time:

(38) $$\begin{array}{l} \kappa_{1}\left(n,m=0\right)=0,\;\kappa_{2}\left(n,m=0\right)=0\\ \kappa_{1}\left(n=0,m\right)=0,\;\kappa_{2}\left(n=0,m\right)=0, \end{array}$$

finally resulting in κ_1_(*n, m* + 1) and κ_2_(*n, m* + 1). In order to find the proper relation between spatial and temporal discretization, we consider the equation for the velocity at the tip in Equation (37), in which case *N*(*m*) = *n*, and recalling that $\frac{ds}{dt}=v$ yields the relation:

(39) $$\begin{array}{l} ds=L_{gz}\dot{E}dt. \end{array}$$

### 3.3. Implementing Growth

As discussed in section 2.2, growth is implemented via a material derivative with a local growth rate described in Equation (6), representing the elongation of cells in the growth zone, creating a one-dimensional growth flow within the organ. When cells reach a certain threshold size, they stop elongating, thus leaving the size of the growth zone *L*_gz_ constant. Since the total length of the organ increases over time, in the numerical scheme, we add a new segment *ds* at the tip at each time step:

(40) $$\begin{array}{l} N\left(m\right)=N\left(0\right)+m, \end{array}$$

where N(m) is the total number of segments in the organ at time step m, and therefore the total length is *L*(*m*) = *N*(*m*) · *ds*. This is not to be confused with accretive growth, where material is added at the tip alone. Special care is required in assigning the correct curvature values to the newly added segments. At time *m* − 1, we initialize the next *N*(*m*)-th segment so that κ_1_(*N, m* − 1) = 0, κ_2_(*N, m* − 1) = 0, $\vec{\Delta }\left(N,m-1\right)=0$, and *v*(*N, m* − 1) = *L*_*gz*_$\dot{E}$ (the velocity at the tip as defined in Equation 6). Substituting these values in Equations (35) and (36) yields κ_1_(*N, m*) = κ_1_(*N* − 1, *m* − 1) and κ_2_(*N, m*) = κ_2_(*N* − 1, *m* − 1), i.e., the curvature of the new segment is identical to that of its predecessor.

### 3.4. Simulation Parameters

In the simulations presented in the next section, the initial conditions include a straight vertical organ κ(*s, t* = 0) = 0 [i.e., κ_1_(*s, t* = 0) = 0 and κ_2_(*s, t* = 0) = 0], with an initial length *L*_0_ = 1.0 and a growth zone *L*_*gz*_ = 1.0. Boundary conditions are defined with a clamped base κ(*s* = 0, *t*) = 0 [κ_1_(*s* = 0, *t*) = κ_2_(*s* = 0, *t*) = 0]. The organ radius is *R* = 0.1, the proprioceptive coefficient is γ = 0.01, and the tropic sensitivity (when applicable) was taken to be either λ_0_ = 0.1 or λ_1_ = 0.05. The ratio of the proprioceptive and tropic sensitivity values substituted in Equation (17) correspond to balance numbers *B* = 10 and *B* = 5 accordingly, both of which are in the range of what has been observed in plants (Bastien et al., 2013). The maximal curvature is κ_max_ = λ_0_/γ = 10, yielding κ_max_*R* = 1. This means that κ*R* ≤ 1 throughout the simulations, in agreement with the low curvature assumption. The simulation time step is *dt* = 0.1, and the length of the discrete elements is *ds* = 0.01. A constant growth rate was taken all along the growth zone following Equation (39): $\dot{E}$ = *ds*/*dtL*_*gz*_ = 0.1. In the next section, we discuss simulations of specific cases. The code is freely available at https://github.com/poratamir/3D-growth-dynamics.

## 4. Case Examples and Simulations

Here, we discuss various representative cases of internal and external cues. Since the differential growth term is the driver of the dynamics, it is the only term that needs to be defined accordingly. We present the specific form of the differential growth vector for each case, as well as a snapshot of a numerical simulation. Videos of the full simulation dynamics can be found in the Supplementary Material, also showing the evolution of κ and ϕ over time. We note that in the following descriptions, the local response vectors do not necessarily reside in the local cross-section plane. However, this does not change the dynamics, in which the differential growth vector is being projected to the local cross-section plane (see Equations 9, 24, and 25).

### 4.1. Infinitely Distant Constant Stimulus

The simplest type of stimulus is a constant stimulus placed at infinity. In this case, the stimulus is a parallel vector field originating from direction $\hat{n}$ and is constant in space and time, i.e., $\vec{\lambda }=\lambda_{0}\hat{n}$:

(41) $$\begin{array}{l} \vec{\Delta }\left(s,t\right)=\lambda_{0}\hat{n}-\gamma \kappa \left(s,t\right)\hat{N}\left(s,t\right). \end{array}$$

**Figure 6:**
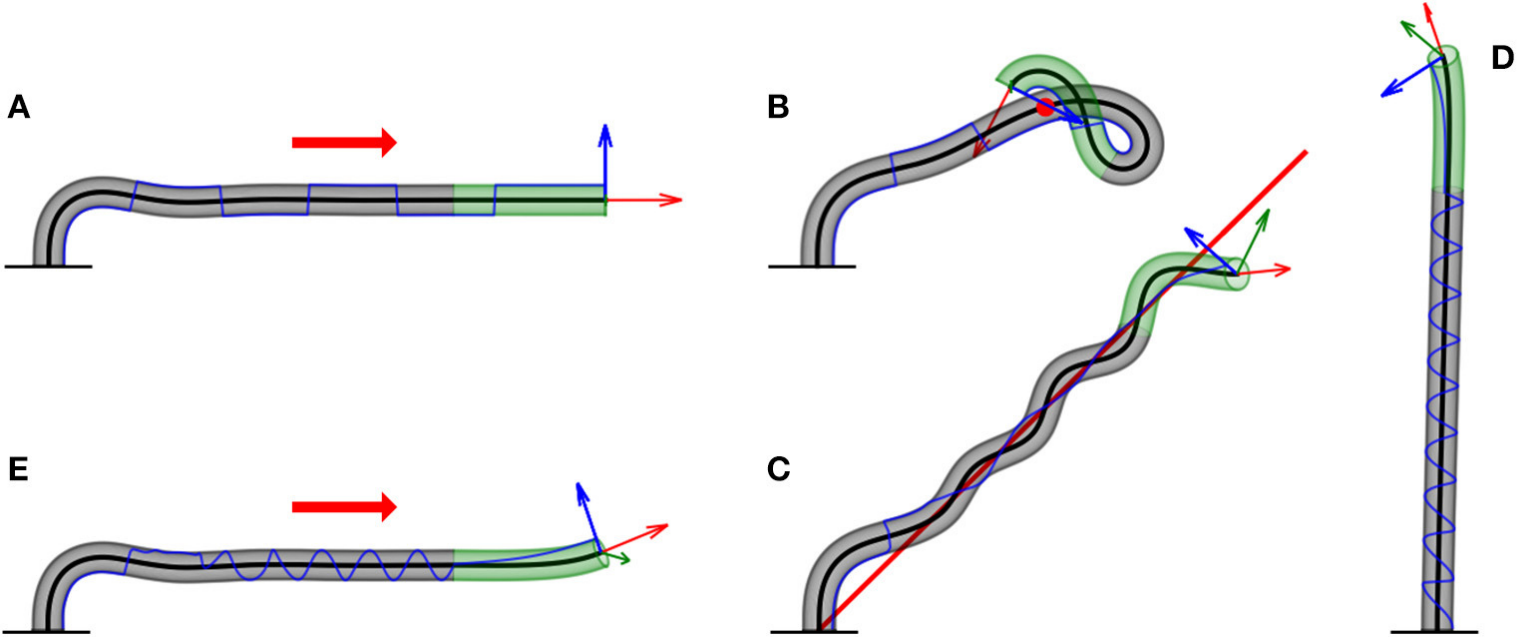
Examples of numerical simulations for various scenarios. Here, we showcase snapshots of simulations for various cases. The subapical active growth zone is in green, while no growth occurs below that in gray. The arrows on the apex are the apical tangent direction $\hat{T}$ (red), normal direction $\hat{N}$ (blue), and bi-normal direction $\hat{B}$ (green). The blue line marks the history of the direction of $\hat{N}$ along the organ. The details of the simulations are given in section 3. We note that elasticity is not implemented here, and therefore the organ grows through itself. **(A)** Infinitely distant constant stimulus (red arrow). The organ reaches a steady state, growing in the direction of the stimulus. $\hat{N}$ switches directions due to damped oscillations in the solution (Video 1). **(B)** Point stimulus (red dot). Illustrates the different dynamics between a distant vs. nearby stimulus (Video 2). **(C)** General geometry: twining around a line stimulus (red line). Any geometry for the source stimulus can be implemented. Here, we chose a line geometry, which, together with a signal in the direction of the line (to prevent self-intersections), yields dynamics similar to the twining of a climbing plant (Video 3). **(D)** Circumnutations. We implement the growth response to an internal cue rather than external cues, yielding inherent periodic movements of plants called circumnutations, generally associated with search processes. The periodic trajectory of $\hat{N}$ visualizes the rotational movement of the growing tip (Video 4). **(E)** Superposition of internal and external stimuli. We combine circumnutations with an infinitely distant external stimulus (Video 5).

The sensitivity λ_0_ may depend on the intensity of the stimulus, for example, in the case of phototropism, following either the Weber Fechner or Stevens' Law, as discussed in section 2.3. This is not the case for gravitropism, since plants sense inclination rather than acceleration (Chauvet et al., 2016). A snapshot of the final form of the simulation is shown in Figure 6A, and an example of the full dynamics can be found in Video 1. Since the projection of this equation in 2D yields the ACE model (Bastien et al., 2013, 2014), we validate our model numerically, showing that our 3D simulations converge to the known analytical solution in 2D with an exponential growth profile (see Appendix B for details).

### 4.2. Point Stimulus

We consider the case of a stimulus whose source is a point located at ${\vec{r}}_{p}$ (Bastien et al., 2019), such as a nearby localized light or water source. In this case, the stimulus leads to a radial vector field centered at the point, i.e., $\vec{\lambda }=\lambda_{0}\frac{{\vec{r}}_{p}-\vec{r}\left(s,t\right)}{\left|{\vec{r}}_{p}-\vec{r}\left(s,t\right)\right|}$:

(42) $$\begin{array}{l} \vec{\Delta }\left(s,t\right)=\lambda_{0}\frac{{\vec{r}}_{p}-\vec{r}\left(s,t\right)}{\left|{\vec{r}}_{p}-\vec{r}\left(s,t\right)\right|}-\gamma \kappa \left(s,t\right)\hat{N}\left(s,t\right). \end{array}$$

Here again, λ_0_ is constant in space; however, this can be generalized to depend on space, for example, in the case of a diffusive chemical where $c\left(\left|{\vec{r}}_{p}-\vec{r}\left(s,t\right)\right|\right)$. A snapshot of the dynamics is shown in Figure 6B, while the full dynamics can be found in Video 2.

### 4.3. General Stimulus Geometry: *Twining* Around a Line Stimulus

We can generalize the point stimulus to any geometrical form. Here, we show an example of a stimulus in the form of an attracting straight line. Let us assume that the line is parallel to an arbitrary direction $\hat{n}$ whose base position in the x-y plane is ${\vec{r}}_{\text{line}}=\left(x_{0},y_{0},0\right)$. The shortest vector between a point on the organ $\vec{r}\left(s,t\right)$ and the line is:

(43) $$\begin{array}{l} \vec{\rho }\left(s,t\right)=\left(\vec{r}\left(s,t\right)-{\vec{r}}_{\text{line}}\right)-\hat{n}\left(\left(\vec{r}\left(s,t\right)-{\vec{r}}_{\text{line}}\right)\cdot \hat{n}\right). \end{array}$$

The response vector will then be $\vec{\lambda }=-\lambda_{0}\hat{\rho }\left(s,t\right)$. As an example of multiple stimuli, we also add a directional stimulus parallel to the line (i.e., gravity or light), $\vec{\lambda }=\lambda_{0}\hat{z}-\lambda_{1}\hat{\rho }\left(s,t\right)$, leading to the following differential growth vector:

(44) $$\begin{array}{l} \vec{\Delta }\left(s,t\right)=\lambda_{0}\hat{Z}-\lambda_{1}\hat{\rho }\left(s,t\right)-\gamma \kappa \left(s,t\right)\hat{N}\left(s,t\right), \end{array}$$

where $\hat{\rho }=\vec{\rho }/|\vec{\rho }|$. The resulting dynamics are reminiscent of the twining motion typical of climbing plants, as shown in Figure 6C and Video 3. We note that this twining movement is not based on touch, meaning that the organ does not hold the support. Furthermore, no elasticity is involved at this stage, as further discussed in the Discussion section.

### 4.4. Internal Processes: Circumnutations

Circumnutations are circular periodic movements of the tips of plant organs, generally associated with search processes, for example, climbing plants searching for a support or roots searching for nutrients. Unlike tropisms, these are inherent movements due to internal drivers, not external stimuli, and can be described as $\vec{\chi }\left(s,t\right)=\lambda_{0}\left(\text{cos }\left(\psi \left(t\right)\right){\hat{m}}_{1}\left(s,t\right)+\text{sin }\left(\psi \left(t\right)\right){\hat{m}}_{2}\left(s,t\right)\right)$, where λ_0_ is the intensity of the bending, ψ(*t*) is a general function describing the direction of growth at time *t*, and we described the direction of growth using the natural frame. Here, we chose a circular form; however, more elaborate forms can be used (Bastien and Meroz, 2016). Following Bastien and Meroz (2016), we substitute $\vec{\chi }\left(s,t\right)$ into the differential growth vector:

(45) $$\begin{array}{l} \vec{\Delta }\left(s,t\right)=\lambda_{0}\left(\text{cos }\left(\psi \left(t\right)\right){\hat{m}}_{1}\left(s,t\right)+\text{sin }\left(\psi \left(t\right)\right){\hat{m}}_{2}\left(s,t\right)\right)\\ \begin{array}{l} \;-\gamma \kappa \left(s,t\right)\hat{N}\left(s,t\right). \end{array} \end{array}$$

In our simulations, we took ψ(*t*) = ω*t* with ω = 0.2/*dt*. A snapshot is found in Figure 6, and the full dynamics can be found in Video 4. The trajectory of $\hat{N}$ clearly illustrates the circular movement of the tip over time.

### 4.5. Superposition of Internal and External Stimuli

As already suggested in the example of a line stimulus, where a directional stimulus is added, we can consider multiple types of stimuli by assuming that they are additive. We present here another example based on plant behavior, where we consider an organ responding to a distant external signal while also exhibiting internally driven circumnutations. In this case, we simply add to Equation (45) the term for the distant stimulus in direction $\hat{n}$, $\lambda_{0}\hat{n}$, yielding:

(46) $$\begin{array}{l} \vec{\Delta }\left(s,t\right)=\lambda_{0}\hat{n}+\lambda_{1}\left(\text{cos }\left(\psi \left(t\right)\right){\hat{m}}_{1}\left(s,t\right)+\text{sin }\left(\psi \left(t\right)\right){\hat{m}}_{2}\left(s,t\right)\right)\\ \begin{array}{l} \;-\gamma \kappa \left(s,t\right)\hat{N}\left(s,t\right) \end{array} \end{array}$$

A snapshot of the resulting dynamics is shown in Figure 6E, and the full dynamics are shown in Video 5.

## 5. Example of an Optimal Control Approach

In this last section, we take a step back and consider a simple example illustrating the possible use of control theory to recover tropic dynamics—in a way that may be amenable for robotics use. In what follows, we no longer use the Frenet Serret formalism developed in this paper, relaxing the assumption of a constant arc-length parameterization. Instead, we consider the general case where the curve of the organ is parameterized using the Lagrangian coordinate *S*_0_, as described in section 2.2, without further reparameterizing the curve as it evolves over time. This general case may be pertinent to some robotics systems. We consider an organ with apical sensing, a fixed length L (neglecting an explicit account for growth, as discussed before), and dynamics restricted to 2D, similar to the case of apical sensing discussed in Bastien et al. (2013). The aim is to find a controlled evolution equation of the tangent $\hat{T}\left(L,t\right)$ at the tip, where $\hat{T}=\partial \vec{r}/\partial S_{0}$. Let $\vec{u}\left(t\right)=\left(u_{1}\left(t\right),u_{2}\left(t\right)\right)$ be a control to orient the tangent at the tip $\hat{T}\left(L,t\right)$. The sensing occurring at the tip influences the dynamics at any other point on the organ, and therefore $\hat{T}\left(s,t\right)$ will satisfy the following Cauchy problem for any s:

(47) $$\{\begin{array}{c} \frac{d}{dt}\hat{T}(s,t)=\int_{0}^{s}\vec{u}(t)ds^{\prime }=s\vec{u}(t),\\ \hat{T}(s,0)={\hat{T}}_{0}(s). \end{array}$$

We further limit the family of possible control strategies to those for which:

(48) $$\begin{array}{l} U=\left\{\vec{u}\in {\mathbb{R}}^{2}\;|\;\hat{n}\cdot \vec{u}\geq 0\right\}, \end{array}$$

where $\hat{n}$ is the direction of the stimulus, since $\hat{n}\cdot \vec{u}\leq 0$ leads to undesired curling dynamics. From these strategies, we wish to choose those that are optimal in some sense. We therefore require that the optimal strategy minimizes some cost function that may manifest some physical element of the robot. Here, we choose the following:

(49) $$\begin{array}{l} W\left(\hat{T},\vec{u}\right)=\int_{0}^{T_{f}}{\left(\hat{T}\left(L,t\right)\cdot \vec{u}\left(t\right)\right)}^{2}dt. \end{array}$$

In this case, the cost function has a geometric meaning: when the dot product goes to zero, together with Equation (47), we have $\dot{\hat{T}}\left(L,t\right)⊥\hat{T}\left(L,t\right)$, i.e., $\left\|\hat{T}\left(L,t\right)\right\|$ is constant, thus recovering the assumption at the basis of the Frenet-Serret formalism of identical parametrization of the arc length over time. This gives a family of optimal controls:

(50) $$\begin{array}{l} {\vec{u}}_{*}\left(t\right)=u_{N}\left(t\right)\hat{N}\left(L,t\right), \end{array}$$

where $\hat{N}\left(L,t\right)$ is the normal direction of the apex, and $u_{N}\left(t\right)\text{sign}\left(\hat{N}\left(L,t\right)\cdot \hat{n}\right)\geq 0$ to satisfy Equation (48). These solutions ensure that the tip approaches the stimulus in a strictly decreasing manner. Indeed, if the initial tangent ${\hat{T}}_{0}\left(L\right)$ is not parallel to $\hat{n}$, then

(51) $$\begin{array}{l} \frac{1}{2}\frac{d}{dt}\|\hat{T}\left(L,t\right)-\hat{n}{\|}^{2}=\left(\hat{T}\left(L,t\right)-\hat{n}\right)\cdot \frac{d}{dt}\hat{T}\left(L,t\right)=-u_{N}\left(t\right)\hat{N}\left(L,t\right)\\ \begin{array}{l} \;\cdot \hat{n}<0 \end{array} \end{array}$$

for all *t*, such that $\hat{T}\left(L,t\right)$ is not parallel to $\hat{n}$. Then, $\hat{T}\left(L,t\right)$ remains constantly parallel to $\hat{n}$. In particular, such a computation implies that the tangent $\hat{T}\left(L,t\right)$ does not oscillate around the stimulus direction $\hat{n}$. We focus our attention to a member of the control family described in Equation (50):

(52) $$\begin{array}{l} {\vec{u}}_{*}\left(t\right)=\beta {\hat{T}}_{*}^{⊥}\left(L,t\right)\left(\hat{n}\cdot {\hat{T}}_{*}^{⊥}\left(L,t\right)\right), \end{array}$$

for all *t* ∈ [0, *T*_*f*_] and β ≥ 0, where ${\hat{T}}_{*}^{⊥}=\left(-T_{2*},T_{1*}\right)=\pm \hat{N}$ is just the vector perpendicular to ${\hat{T}}_{*}=\left(T_{1*},T_{2*}\right)$. In Appendix C, we show the details of the calculation based on Pontryagin's maximum principle (Aronna et al., 2017), showing that this indeed meets the requirements of the optimal control problem described in Equations (47)–(49). Substituting the specific solution described in Equation (52) into the dynamics of Equation (47) while also writing the tangent vector in terms of the angle θ(*s, t*) between $\hat{T}\left(s,t\right)$ and the stimulus direction $\hat{n}$, i.e., $\hat{T}\left(s,t\right)=\left(\text{sin }\theta \left(s,t\right),\text{cos }\theta \left(s,t\right)\right)$, yields the following dynamical equation:

(53) $$\begin{array}{l} \frac{\partial }{\partial t}\frac{\partial }{\partial s}\theta \left(s,t\right)=-\text{sin }\theta \left(L,t\right), \end{array}$$

which is identical to the dynamics described in Bastien et al. (2013) in the case of apical sensing, where proprioception is not required for stability.

## 6. Discussion and Conclusion

In this work, we presented a general and rigorous mathematical framework of a rod-like growing organ whose dynamics are driven by a differential growth vector. We constructed the differential growth vector by taking into account both internal and external cues, as well as posture control, as schematically illustrated in Figure 5. The model adopts the 3D Frenet-Serret formalism, which is a natural choice to describe curves and is useful for robotics control purposes. In recent years, there has been an advancement in the mathematical description of plant growth-driven movements, as described in the Introduction. A careful comparison of our model to previous models finds that our model is general, consolidating different aspects in 3D for the first time: growth-driven responses to both external and internal cues, allowing stimuli with different physical and geometrical characteristics while maintaining posture control through proprioception.

We ran numerical simulations of a number of key cases. In the case of the response to external stimuli, we considered a distant stimulus (such as sunlight and gravity), a point stimulus (such as a point light source), and a rod stimulus that emulates *twining* of a climbing plant around a support. We also simulated circumnutations, the response to an internal oscillatory cue associated with search processes. Lastly, we also demonstrated the superposition of the response to an external stimulus and circumnutations. These examples showcase the broad spectrum of cases that this framework can describe and represent interactions with the environment, which are at the basis of the autonomous performance of next-generation self-growing robots in unstructured scenarios, including movement in uncertain terrains involving obstacles and voids. The model presented here therefore establishes the basis for a control system for robots with a changing and unpredictable morphology.

While building a physical robotic representation that can behave as the model predicts is well out of the realm of current technology, the current model can be simplified so as to be relevant for current technologies, yielding limited behavior. As an example, current additive manufacturing technologies are generally limited to the addition of material at the tip, with no elongation. This accretive growth can be represented in our model by taking the growth zone to an infinitesimal size. In order to account for a robotic structure made of a number of rigid components with hinges, nodes, etc., the infinitesimal segments *ds* can be taken to be finite. Another example concerns the sensory system of the robot, whose characteristics can be readily represented in the model. In other words, the model is general enough to capture the essence of a variety of different robotic capabilities, which is particularly important in an era of quickly developing technologies.

Following this line of thought, we note that the framework presented here disregards parameters pertinent to robotic structures, such as energy, friction, weight, etc. In this paper, we present a simple example illustrating the possible use of optimal control theory in order to recover tropic dynamics in a way that may be relevant for robotics use. Optimal control theory optimizes processes where some cost function is minimized, and it is therefore useful in engineering problems. The example *per se* does not necessarily present a practical cost function; however, it suggests that future work may include optimizing the current model for tropic movements so as to minimize a cost function associated with a robotic parameter.

This general framework allows a deeper understanding of plant dynamics in response to their environment. Indeed, while current investigations on tropisms are generally restricted to 2D, our model enables the quantitative study of tropisms in 3D, i.e., where single or multiple stimuli are placed outside of the organ plane. Furthermore, careful attention has been paid to relating environmental stimuli to differential growth, discussing stimuli with different physical characteristics categorized by their mathematical description, such as vector fields (light and gravity), and scalar fields (concentration of water or nutrients). Indeed, the latter finally allows a rigorous characterization of plant biosensors in tropisms that are less understood, such as hydrotropism and chemotropism, as well as a currently lacking quantitative analysis of their dynamics.

Understanding plant movements is essential for a rigorous understanding of plant behavior—a field that has only recently become the focus of research. Basic behavioral processes in animals are generally studied through their motor responses to controlled stimuli, and a solid understanding of plant movements (in response to both internal and external cues) paves the way to designing controlled behavioral experiments. For example, simulations incorporating both circumnutations and tropisms will allow quantitative investigation of the role of circumnutations in the successful search for nutrients or light.

Though the framework we develop here successfully describes various scenarios of growth-driven movements of plants, it of course differs from its botanical inspiration. One main difference is that here we do not consider branching. Furthermore, as noted throughout the text, this framework does not currently include mechanics or elasticity, disregarding any elastic responses of the organ to physical forces. However, this can be naturally implemented in the Frenet-Serret frame of reference (Chelakkot and Mahadevan, 2017; Goriely, 2017; Agostinelli et al., 2020), which we plan to pursue, together with branching, in future work. On the other hand, we note that our model is general enough so that it can be customized to represent a specific biological system, e.g., by changing the growth profile of a growth zone or the geometry of the sensory system. Furthermore, note that though this framework is inspired by plant responses, it is not based on biological details and is therefore amenable to any rod-like organisms that respond to signals via growth, such as neurons and fungi.

## Data Availability Statement

The numerical code for simulations presented in this work is accessible at: https://github.com/poratamir/3D-growth-dynamics.

## Author Contributions

AP and YM developed the mathematical framework. AP carried out the numerical analysis and simulations and prepared all the figures and videos. FT, MP, and PM developed the optimal control calculation and the comparison with the model by Bressan et al. AP and YM drafted the manuscript. All authors contributed to the authoring of the final manuscript and contributed to the research design.

## Conflict of Interest

The authors declare that the research was conducted in the absence of any commercial or financial relationships that could be construed as a potential conflict of interest.

## Supplementary Material

The supplementary material includes one pdf file and 5 videos.

Supplementary Data Sheet 1 Includes three Appendices:

Appendix A Derivation of growth dynamics both in the Frenet-Serret frame and the natural frame, relevant to sections 2.2 and 3.

Appendix B Shows that our 3D simulations converge to the known analytical solution in 2D with an exponential growth profile.

Appendix C Pontryagin's Maximum Principle, part of the calculation in the optimal control approach in section 5.

The videos show simulations for the different cases presented in section 4, and Figure 6 shows snapshots of the simulations. The arrows represent the Frenet-Serret Frame ($\hat{T}$ in red, $\hat{N}$ in blue, and $\hat{B}$ in green). The graphs show the values of the curvature κ(*s, t*) and the angle ϕ(*s, t*) as a function of time:

Video 1 Infinitely distant constant stimulus.

Video 2 Point stimulus.

Video 3 General stimulus geometry: twining around a line stimulus.

Video 4 Internal processes: circumnutations.

Video 5 Superposition of internal and external stimuli.
